# Elevated CD47 Expression Impairs Elimination of Photoaged Fibroblasts by Macrophages and Serves as a Potential Biomarker for Photoaging

**DOI:** 10.1111/jocd.70098

**Published:** 2025-04-09

**Authors:** Xinya Xu, Xinhua Lu, Xinling Chen, Amin Yao, Wei Lai

**Affiliations:** ^1^ Department of Dermatology The Third Affiliated Hospital of Sun Yat‐Sen University Guangzhou China; ^2^ Department of Neurosurgery Foresea Life Insurance Guangzhou General Hospital Guangzhou China

**Keywords:** Fibroblasts, Photoaging, UVA

## Abstract

**Background:**

CD47 could negatively regulate macrophage‐mediated phagocytosis and contribute to senescent cells accumulation in aging. However, it remains unknown whether CD47 is overexpressed in photoaged skin and involved in photoaging pathogenesis.

**Aims:**

To investigate the expression, clinical significance, and mechanism of CD47 in photoaging.

**Methods:**

Sun‐exposed (*n* = 10) and sun‐protected (*n* = 10) skin samples were collected from elderly subjects and stained for CD47, and its association with collagen and elastin content and p16 expression was subsequently analyzed. A cellular photoaging model was then established to examine CD47 expression in photoaged fibroblasts. Furthermore, the influence of photoaged fibroblasts on macrophage‐mediated phagocytosis and elimination was assessed by constructing a co‐culture system. SiRNA was applied to block the CD47/SIRPα axis to determine its role in this process. Finally, the activation of the CD47/SIRPα axis was evaluated in skin samples.

**Results:**

We showed the increased dermal CD47 expression in sun‐exposed aged skin, which was closely correlated with the reduced collagen content and enhanced elastin accumulation and dermal p16 expression. Next, elevated CD47 was detected in both sun‐exposed aged skin‐derived fibroblasts and photoaged ones. We discovered that photoaged fibroblasts impaired the phagocytotic function of co‐cultured macrophages via CD47/SIRPα axis, and blocking the CD47/SIRPα axis could improve their elimination. Moreover, the CD47/SIRPα axis was found to be activated in the sun‐exposed aged skin.

**Conclusions:**

The present study demonstrated for the first time that CD47 was highly expressed and involved in mediating photoaged fibroblasts accumulation, providing important evidence for CD47 as a potential biomarker and therapeutic target for photoaging.

## 1 Introduction

The skin is the largest organ of the human body, acting as a crucial barrier between the internal and external environments. Constant exposure to endogenous and exogenous stresses leads to skin aging, which is mainly divided into intrinsic and extrinsic aging (photoaging) [1]. Since photoaging reportedly accounts for approximately 80% of skin aging [1] and contributes to photo‐inflammation and even photo‐carcinogenesis [2], research on photoaging has always been a global priority. Chronic ultraviolet radiation (UVR), mainly involving UVA (320–400 nm) and UVB (280–320 nm), is generally considered as the key inducer of photoaging. Specifically, UVB generally acts on the epidermal layer and induces sunburn, while UVA could deeply penetrate into the dermis causing disorganization of connective tissue [1]. Various mechanisms including cell senescence, DNA damage, oxidative stress, and inflammation, are involved in the process of UVR‐induced skin photoaging [1, 2, 3]. Nevertheless, the mechanisms underlying photoaging pathogenesis are still not entirely elucidated.

Senescent cells accumulate with age occurs in virtually all tissues and organs, serving as a well‐established hallmark of aging [4]. As the primary cell component of dermal connective tissue, fibroblasts are more susceptible to undergo senescence under chronic UVA radiation. Of note, the accumulation of senescent fibroblasts during photoaging has been suggested by the increased number of p16 positive fibroblasts observed in the sun‐exposed aged skin [5]. Interestingly, appropriate accumulation of senescent fibroblasts plays beneficial roles in physiological processes such as wound repair [6]. However, once the subsequent immune clearance is broken, the persistently accumulated senescent fibroblasts become detrimental. The pathological roles of UVA‐induced senescent (photoaged) fibroblasts have been well established over the years. It is known that photoaged fibroblasts exhibit abnormally activated matrix metalloproteinases (MMPs) and diminished capacity of collagen synthesis, leading to reduced dermal collagen contents [7, 8]. Moreover, the elafin generated by photoaged fibroblasts has been validated to bond with elastic fibers, rendering them resistant to enzymatic proteolysis and excessive accumulated [9]. Additionally, our previous work identified the dysregulated cathepsins in photoaged fibroblasts, which also contribute to the decreased degradation of elastic fibers [10]. Furthermore, the senescence‐associated secretory phenotype (SASP) factors derived from photoaged fibroblasts have been highlighted recently, which are implicated in eliciting chronic low‐grade inflammation and even immunosuppression [11]. Strikingly, Kim et al. [12] reported that the senescent fibroblasts were remarkably accumulated in the skin tissues of photoaging mouse model, and pharmacologically eliminating the senescent fibroblasts could dramatically alleviate the skin photoaging phenotype. Therefore, it would be therapeutically beneficial for photoaging to prevent senescent fibroblast accumulation. However, the mechanism underlying this accumulation remains poorly understood and warrants further study.

Macrophages, with high phagocytic activity, are key players in eliminating senescent cells to maintain tissue homoeostasis [13]. Macrophage‐mediated phagocytosis is generally initiated by recognizing pro‐phagocytic “eat me” signals, like phosphatidylserine, exposed on target cells, but this can be counterbalanced by anti‐phagocytic “don't eat me” signal [14]. It has been established that senescent cells are able to manipulate the “don't eat me” signals to evade phagocytosis by macrophages [13]. CD47 is one of the most frequently reported “don't eat me” signals hijacked by senescent cells, which transmit inhibitory signals to macrophages through direct contacting with signal‐regulatory protein alpha (SIRPα) [13]. Recently, CD47/SIRPα axis has been reported to be involved in senescent cells accumulation in various age‐related disorders, such as bone loss [15] and sarcopenia [16]，and this axis is becoming a promising target for therapeutic intervention. Increased CD47 expression has been observed in the chemical‐induced senescent fibroblasts [17]. However, whether CD47 expression is elevated in fibroblasts undergoing photoaging remains to be determined. In addition, Horiba et al. [18] identified M1‐type macrophages, characterized by weak phagocytic function, as the mainly phenotype of macrophages infiltrated in sun‐exposed aged skin. Whether CD47 negatively regulates macrophage‐mediated phagocytosis and thereby leads to senescent fibroblasts accumulation during photoaging is also unclear.

Here, we showed increased CD47 expression in the dermis of sun‐exposed aged skin, which was strongly associated with collagen degradation, elastic fibers accumulation, and dermal p16 expression. By establishing a photoaging cellular model, we confirmed that the photoaged fibroblasts overexpressed CD47 to evade macrophage‐mediated elimination upon binding with SIRPα. Furthermore, the CD47/SIRPα axis was demonstrated to be activated in the sun‐exposed aged skin. Through the present study, we not only revealed the mechanism underlying senescence fibroblasts accumulation during photoaging, but also validated the potential role of CD47 as a novel biomarker for assessing photoaging.

## 2 Methods

### 2.1 Patients and Clinical Samples

Skin samples were obtained from peripheral skin after the benign skin tumorectomy conducted in our department. The ethics approval of the present study was obtained from the committee of our institution. Signed informed consent was obtained from each patient before enrollment. This study was conducted in strict accordance with the Declaration of Helsinki. According to the original location, the collected skin samples were divided into the sun‐exposed (*n* = 10; mean age, 72.1 years; age range, 61–83 years; female: male = 4:6) and sun‐protected (*n* = 10; mean age, 70.7 years; age range, 63–81 years; female: male = 4:6) groups. For subsequent experiments, these fresh skin samples were fixed with formalin, paraffin‐embedded, and sectioned for the subsequent experiments.

### 2.2 Immunohistochemistry

For immunohistochemistry, the sections were subjected to deparaffinized hydrated, and pretreated for antigen retrieval with citrate buffer (0.01 M, PH 6.0). Following blocking, the sections were incubated overnight at 4°C with the primary antibodies against CD47 (1:1000; 20 305‐1‐AP, Proteintech, Chicago, USA) and p16 (1:500; AF5484, Affinity, OH, USA). After incubation with biotinylated secondary antibody (1:500; SA00004‐2, Proteintech) for 30 min at room temperature, AEC chromogen solution (Biozol, Eching, Germany) was applied for visualization. Finally, the sections were observed under a microscope and photographed for analysis with ImageJ software (National Institute of Health, Bethesda, USA).

### 2.3 Immunofluorescence Staining

The skin sections for immunohistochemistry were processed in the same manner as described above. As for the cultured fibroblasts and macrophages, they were formaldehyde‐fixed and Triton‐X‐100 permeabilized after the indicated treatment. After blocking with 3% BSA, tissue sections or cell samples were incubated overnight at 4°C with diluted primary antibodies, including anti‐CD47 (1:200; Proteintech), anti‐p16 (1:200; Affinity), anti‐Vimentin (1:500; 60 330‐1‐lg, Proteintech), anti‐SIRPα (1:50; A9001, ABclonal, China) and anti‐CD68 (1:50; AB955, Abcam, Cambridge, UK). On the next day, samples for single or double color staining were incubated with species‐matched fluorescently labeled secondary antibodies (Alexa Fluor 488‐conjugated goat anti‐mouse IgG, #4408; Alexa Fluor 594‐conjugated goat anti‐rabbit IgG, #4412, Cell Signaling Technology, MA, USA) for 1 h and then stained with DAPI (1:10000; Beyotime Biotechnology, Shanghai, China). For triple immunofluorescence staining, tyramide signal amplification (TSA) Plus Fluorescence Kits (Perkin Elmer, MA, USA) was used according to the manufacturer's protocol. The immunofluorescent signals were observed and photographed using a fluorescence microscope.

### 2.4 Masson's Trichrome and EVG Staining

The collagen content was detected by Masson's trichrome staining, which performed with Masson trichrome staining kit (Beyotime Biotechnology) according to the manufacturer's protocol after the paraffin sections were dewaxed and rehydrated. Elastica van Gieson (EVG) staining was conducted with an EVG staining kit (Solarbio, Beijing, China) for elastic fiber assessment. Following routine dewaxing and hydration, the sections were placed in elastic stain solution for 30 min, followed by differentiation with a 5% ferric chloride solution. The content of collagen (stained blue) and elastic fibers (stained black) was quantified as a percentage of total tissue area using ImageJ software.

### 2.5 Cell Culture and Treatment

Human dermal fibroblasts were isolated from children's foreskin specimens obtained following circumcision using Dispase II enzyme (Sigma Aldrich, MO, USA). Fibroblasts were cultured in DMEM medium containing 10% FBS (Procell, Wuhan, China) at 37°C with 5% CO_2_. For all experiments, fibroblasts from passages 2–4 were used. The method for cellular photoaging model construction was referred to our previous study [19]. In short, fibroblasts were exposed to 10 J/cm^2^ UVA irradiation once daily for 14 consecutive days using a UVA lamp (Sigma, Shanghai, China). Control fibroblasts were treated identically but without exposure to UVA irradiation. The intensity of radiation was determined by using a UVX digital radiometer (UVP Inc., San Gabriel, CA, USA).

The human promonocytic THP‐1 cell line (CL‐0233, Procell) was cultured in RPMI‐1640 media supplemented with 10% FBS and 1% glutamine (Procell). To differentiate THP‐1 monocytes into macrophages, THP‐1 monocytes were stimulated with 100 nM PMA (Solarbio) for 48 h.

### 2.6 Co‐Culture Model System

Macrophages were co‐cultured in contact with control and photoaged fibroblasts at a ratio of 5:1 for 16 h, respectively, and then the fibroblasts were detached with accutase (ThermoFisher, MA, USA). Simultaneously, apoptosis was induced in the Jurkat cells (CL‐0315, Procell) by using staurosporine (1 μM, 4 h; Beyotime Biotechnology), a classic apoptosis inducer. After labeling with carboxyfluorescein succinimidyl ester (CFSE; 1:1000; Thermo Fisher Scientific, MA, USA), the apoptotic Jurkat cells were further co‐cultured with the macrophages at a 5:1 ratio for 2 h. After washing to remove the un‐engulfed cells, the cells were viewed and photographed under light and fluorescence microscope. To quantify phagocytic activity, the proportion of macrophages engulfed CFSE‐labeled apoptotic Jurkat cells was calculated.

To assess the ability of macrophages to eliminate photoaged fibroblasts, they were co‐cultured at a 5:1 ratio (macrophage: fibroblast). Before co‐culture, the photoaged fibroblasts were also labeled with CFSE. To determine the role of CD47‐SIRPα axis in this process, CD47 siRNA (sc‐35 006, Santa Cruz, CA, USA) and SIRPα siRNA (sc‐44 106, Santa Cruz) were applied to block their expression using Lipo3000 (Invitrogen, CA, USA), respectively. After 48 h of co‐culture, the number of CFSE‐positive cells was counted under a fluorescence microscope.

### 2.7 SA‐β‐Gal Staining

Senescence‐Associated β‐Galactosidase (SA‐β‐gal) staining was applied to validate the senescent phenotype of UVA‐irradiated fibroblasts using a SA‐β‐gal Staining Kit (Beyotime Biotechnology). After the indicated treatment, the cells were washed with PBS and then fixed with fixative solution for 15 min at room temperature. Thereafter, the fixed cells were incubated overnight with SA‐β‐gal staining solution at 37°C. The following day, SA‐β‐gal positive cells (blue‐stained) were observed and counted under a light microscope.

### 2.8 Western Blotting

Cell lysates were prepared using RIPA lysis buffer (Beyotime Biotechnology) and then subjected to protein concentration determination using a BCA assay. Thereafter, equal amounts of protein were separated by SDS‐PAGE and further electrophoretic transferred to polyvinylidene fluoride (PVDF) membranes (Millipore, MA, USA). After blocking with 5% of BSA solution for 1 h, incubation with the primary antibody followed by the corresponding HRP‐conjugated secondary antibody (1:2000; SA00001‐1 and SA00001‐1, Proteintech) were performed. Subsequent detection of immunoprecipitated proteins was conducted using an ECL Detection Kit (Millipore) and imaged on the LAS‐3000 Imaging System (Fujifilm Corporation, Tokyo, Japan). The grayscale values of western blotting bands were analyzed with ImageJ software. The primary antibodies used in this assay were the following: anti‐CD47 (1:1000; Proteintech), anti‐SIRPα (1:1000; ABclonal), anti‐p16 (1:1000; Affinity), anti‐p53 (1:2000; 10 442‐1‐AP, Proteintech), anti‐p21 (1:1000; 10 355‐1‐AP, Proteintech) and anti‐β‐actin (1:2000; 81 115‐1‐RR, Proteintech).

### 2.9 Statistical Analysis

GraphPad Prism version 10 software (GraphPad Software Company, San Diego, CA) was used for statistical analyses. All experiments were performed independently three times, and data were presented as mean ± SEM. Comparison between two groups was performed using an unpaired Student's *t*‐test, while comparison among multiple groups was performed by one‐way ANOVA. For correlation analysis, Spearman's Rank Correlation test was used. *p*‐values < 0.05 were regarded as statistically significant.

## 3 Results

### 3.1 CD47 Is Overexpressed in the Sun‐Exposed Aged Skin Dermis and Associated With Photoaging‐Related Pathological Alterations

**FIGURE 1:**
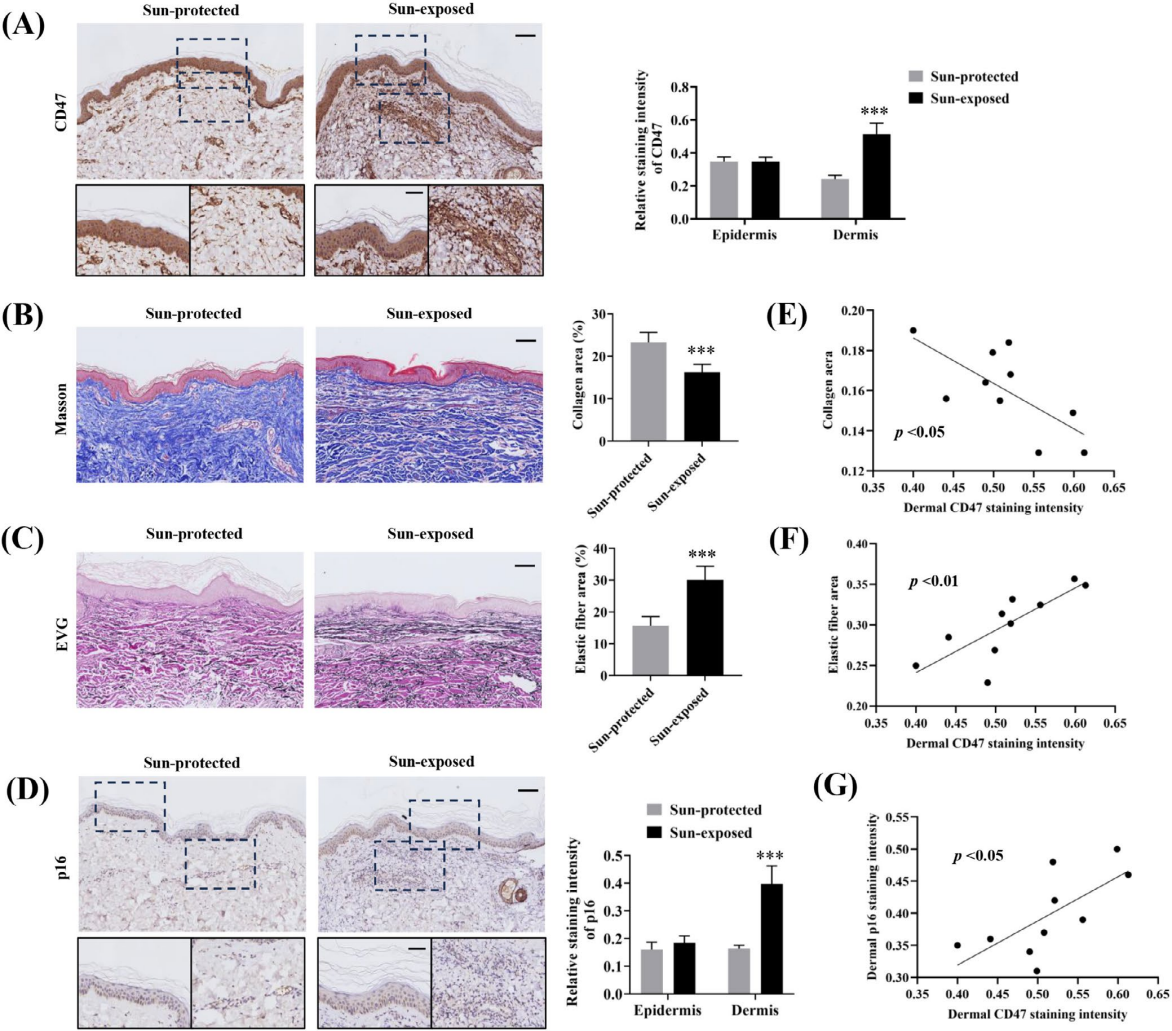
Increased dermal CD47 expression in the sun‐exposed aged skin and its association with collagen content, elastin accumulation, and dermal p16 expression. (A) Representative images and semi‐quantitative histogram of immunohistochemical staining of CD47 in skin samples of sun‐protected (*n* = 10) and sun‐exposed (*n* = 10) groups. Scale bar, 100 μm and 50 μm. (B) Representative images of Masson‐trichrome staining (collagens appear in blue) and the corresponding quantification of collagen deposition. Scale bar, 100 μm. (C) Correlation analysis of dermal CD47 expression with collagen content in the sun‐exposed group. (D) Representative images of Elastica van Gieson (EVG) staining (elastic fibers appear in black) and the corresponding quantification of elastic fibers. Scale bar, 100 μm. (E) Correlation analysis between dermal CD47 expression and elastic fiber content in the sun‐exposed group. (F) Cell senescence marker p16 staining and semi‐quantitative analysis. Scale bar, 100 μm. (G) Correlation analysis between dermal CD47 and p21 expression in the sun‐exposed group. Data are the means ± SD from three independent experiments. ***p < 0.001.

We first examined CD47 expression in sun‐exposed (*n* = 10) and sun‐protected (*n* = 10) skin samples obtained from age‐ and sex‐matched elderly subjects using immunohistochemistry. Figure 1A showed that the dermal and epidermal expression of CD47 in the sun‐exposed group was remarkably higher than that in sun‐protected group. Moreover, the sun‐exposed aged skin also exhibited significantly enhanced dermal CD47 expression compared with the sun‐protected aged skin. However, no such increase was observed for epidermal CD47 expression. These findings suggested that the dermal CD47 expression is likely to be influenced by UV radiation and related to photoaging.

Histopathologically, photoaging is characterized by collagen reduction, elastic fibers accumulation, and abnormal expression of senescence markers, which are also used as quantitative parameters to assess skin photoaging [1, 19]. As expected, Masson trichrome and EVG staining showed that the collagen content was remarkably reduced in the sun‐exposed aged skin (Figure 1B), whereas the elastin accumulation was significantly increased (Figure 1C). Moreover, dermal but not epidermal expression of p16, a widely known senescence biomarker, evaluated by immunohistochemistry staining was significantly increased in the sun‐exposed group (Figure 1D). We next sought to assess any potential association of dermal CD47 expression in relation to the above‐tested hallmarks of photoaging. In the sun‐exposed group, dermal CD47 expression was found to be negatively correlated with collagen content (Figure 1E) and positively correlated with elastin amount (Figure 1F) and dermal p16 expression (Figure 1G). These results suggested that dermal CD47 is closely associated with the pathological changes of photoaging and might serve as a potential biomarker to evaluate photoaging.

### 3.2 CD47 Expression Is Elevated in Photoaged Fibroblasts

**FIGURE 2:**
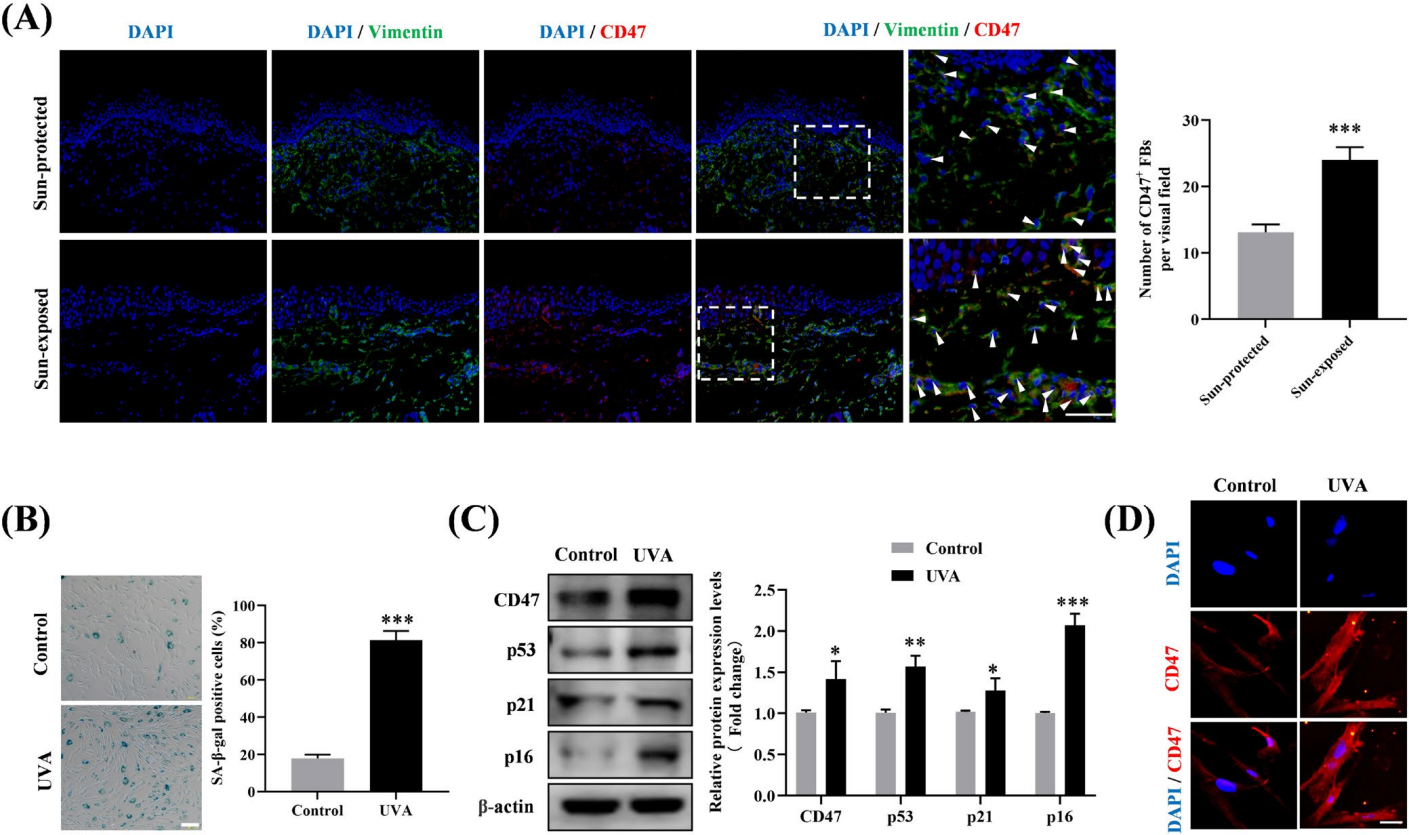
Elevated CD47 expression levels in photoaged fibroblasts. (A) Immunofluorescence double staining showed the location and expression of CD47 in skin tissues of each group. Fibroblasts were labeled using Vimentin (green) and nuclei were stained with DAPI (blue). CD47 was labeled as red. The quantification of CD47/Vimentin double‐positive cells was shown in the right side. Scale bar, 50 μm. (B) Images of SA‐β‐gal staining and quantification of the SA‐β‐gal‐positive rate in UVA‐irradiated fibroblasts. Scale bar, 200 μm. (C) The protein expression levels of CD47, p53, p21, and p16 in control and UVA‐irradiated fibroblasts were detected by Western blotting. (D) Immunofluorescence detection of CD47 protein expression in control and UVA‐irradiated fibroblasts. The red marker indicates cells expressing CD47 and the blue marker indicates DAPI‐stained nuclei. Scale bar, 50 μm. Data are the means ± SD from three independent experiments. *p < 0.05, **p < 0.01, ***p < 0.001. FBs, fibroblasts.

Next, we performed double immunohistofluorescence in the skin samples to describe the cellular localization of CD47. The results showed that CD47 was primarily colocalized with the fibroblast marker Vimentin in both two groups, implying that CD47 was mainly expressed in dermal fibroblasts (Figure 2A). Moreover, the sun‐exposed aged skin also presented a markedly higher number of CD47^+^ fibroblasts than the sun‐protected aged skin (Figure 2A). Hence, we speculated that CD47 expression in fibroblasts may be upregulated by UV radiation.

To verify this speculation, cellular photoaging model induced by repeated UVA exposure was constructed in vitro utilizing human dermal fibroblasts. As demonstrated by SA‐β‐galactosidase staining, chronic UVA radiation (10 J/cm^2^, once daily for 14 consecutive days) led to a considerable rise in the positive rate of SA‐β‐galactosidase in fibroblasts (Figure 2B). Furthermore, UVA‐irradiated fibroblasts displayed significantly enhanced protein expression of cellular senescence markers including p53, p21, and p16 (Figure 2C). These results confirmed the successful establishment of a cellular photoaging model. Furthermore, we observed a concurrent increase in CD47 expression in the photoaged fibroblasts, as evaluated by Western blotting (Figure 2C) and immunofluorescence (Figure 2D). Our findings suggested that chronic UVA radiation contributes to the elevated CD47 expression in fibroblasts during photoaging.

### 3.3 The CD47/SIRPα Axis Hinders Elimination of Photoaged Fibroblasts by Macrophages

**FIGURE 3:**
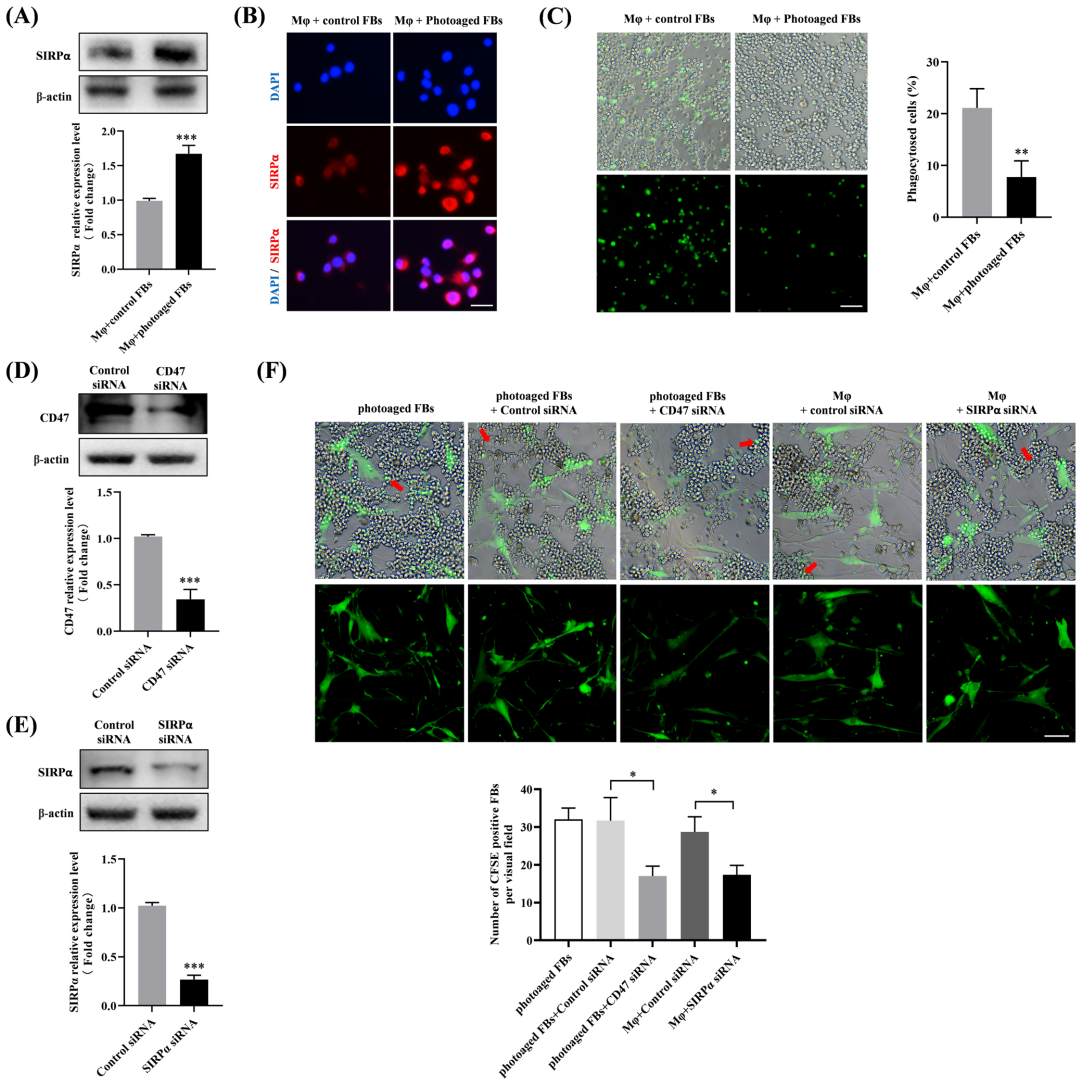
The CD47‐SIRPα axis impairs macrophage‐mediated phagocytosis and blockage of CD47‐SIRPα axis could improve the clearance of photoaged fibroblasts by macrophages. (A) Western blotting for SIRPα expression in macrophages co‐cultured with photoaged fibroblasts. (B) Representative pictures of SIRPα immunofluorescence staining. Red fluorescence for SIRPα, blue fluorescence for DAPI. Scale bar, 25 μm. (C) Macrophages were first co‐cultured with control or photoaged fibroblasts for 16 h and then switched to co‐culture with CFSE‐labeled (green) apoptotic Jurkat cells for 2 h. Representative images and quantified percentages of CFSE‐labeled (green fluorescence) apoptotic Jurkat cells phagocytosed by macrophages in each group. Scale bar, 50 μm. (D) Western blot analysis and quantification analysis of CD47 protein expression in photoaged fibroblasts transfected with CD47 siRNA or control siRNA. (E) Western blot analysis and quantification analysis of SIRPα protein expression in macrophages transfected with SIRPα siRNA or control siRNA. (F) Representative images of the photoaged fibroblasts‐macrophages co‐culture system with indicated treatments and related quantitative analysis of photoaged fibroblasts counts. The green color represents the residual CFSE‐labeled photoaged fibroblasts un‐eliminated by macrophages. Scale bar, 50 μm. Data are the means ± SD from three independent experiments. *p < 0.05, **p < 0.01, ***p < 0.001. FBs, fibroblasts. Mφ, macrophage.

As a “don't‐eat‐me” signaling molecule, CD47 is known to impair macrophage‐mediated phagocytosis of senescent cells through direct binding to SIRPα, resulting in senescent cells accumulation [13]. Given that the accumulation of senescent fibroblasts has been confirmed in photoaging, we therefore wonder whether the CD47/SIRPα axis was involved in this process. To this end, THP‐1 derived macrophages were first co‐cultured in contact with control or photoaged fibroblasts, and then incubated with CFSE‐labeled apoptotic Jurkat cells. We observed significantly upregulated expression of SIRPα in macrophages co‐cultured with photoaged fibroblasts, as measured by Western blotting (Figure 3A) and immunofluorescence (Figure 3B). Moreover, fluorescence microscopy results showed that direct contact with photoaged fibroblasts substantially suppressed the phagocytotic ability of macrophages (Figure 3C). To determine the functional role of the CD47/SIRPα axis in this process, siRNA‐mediated CD47/SIRPα knockdown was conducted in photoaged fibroblasts and macrophages (Figure 3D,E), respectively, before co‐culture. The results showed that the photoaged fibroblasts could be phagocytosed by macrophages (indicated by red arrows), and siRNA‐induced CD47 or SIRPα deficiency could efficiently prompted photoaged fibroblasts to be eliminated by macrophages (Figure 3F). The above data suggested that the CD47/SIRPα activation could inhibit macrophage‐mediated phagocytosis, leading to the impaired elimination of photoaged fibroblasts by macrophages.

### 3.4 The CD47/SIRPα Axis Is Activated in the Sun‐Exposed Aged Skin

**FIGURE 4:**
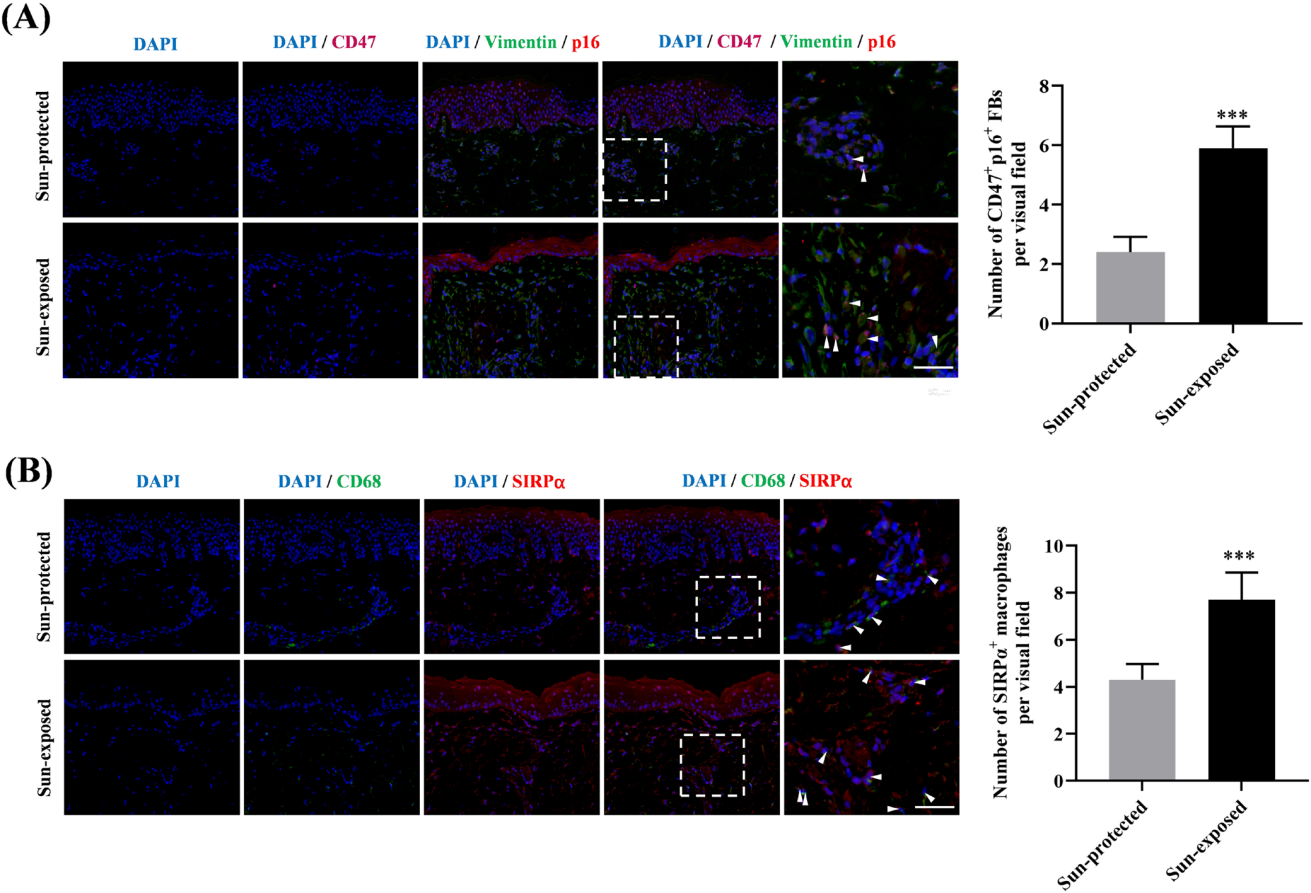
Increased number of CD47^+^ senescent fibroblasts and SIRPα^+^ macrophages in the sun‐exposed aged skin. (A) Triple immunofluorescence staining for CD47 (deep red), Vimentin (green) and p21 (red) in skin tissues of sun‐protected and sun‐exposed groups. Blue: DAPI nuclear counterstaining. Bar graphs represent the number of CD47, p16, and Vimentin triple‐positive cells. Scale bar, 50 μm. (B) Double immunofluorescence staining for CD68 (green) and SIRPα (red) in skin tissues of each group. Blue: DAPI nuclear counterstaining. Bar graphs represent the number of CD68/SIRPα double‐positive cells. Data are the means ± SD from three independent experiments. ***p < 0.001. FBs, fibroblasts. Mφ, macrophage.

Based on these in vitro observations, we attempted to validate our findings in skin samples. By conducting treble immunofluorescence staining, we observed a significantly increased number of senescent fibroblasts (p16/Vimentin double positive cells), as well as CD47^+^ senescent fibroblasts, accumulated in the sun‐exposed aged dermis (Figure 4A). In parallel, the number of SIRPα^+^ macrophages detected in the sun‐exposed aged skin was also obviously increased (Figure 4B). Our results indicated the involvement of the CD47/SIRPα axis in senescent fibroblasts accumulation during photoaging.

## 4 DISSCUSSION

The accumulation of senescent cells is an important hallmark of aging and multiple age‐related diseases could be induced by the increased senescent cell burden [4]. Although the senescent fibroblasts have been shown to accumulate in photoaged skin [5] and involved in the pathogenesis of photoaging [11], the mechanism underlying this accumulation remains unclear. Here, we initially demonstrated that CD47 expression was elevated in the sun‐exposed aged skin dermis, and closely associated with collagen reduction, elastin accumulation and dermal p16 expression. Mechanistically, we verified increased CD47 expression in photoaged fibroblasts, which assisted them in escaping from macrophage‐mediated elimination via binding with SIRPα. Furthermore, the activated CD47/SIRPα axis was confirmed in the sun‐exposed aged skin. The present study uncovered a novel mechanism underlying senescent fibroblasts accumulation in photoaging, and indicated CD47 as a potential biomarker and therapeutic target for photoaging.

Photoaging refers to an accelerated skin aging process triggered by chronic ultraviolet (UV) exposure [1]. Histologically, photoaging is characterized by reduced collagen content and elastic fibers accumulation, which contribute to skin functional and esthetic alterations including rough textured appearance, thick wrinkles and loss of skin elasticity [1]. Photoaged skin also exhibited abnormal expression of senescence markers, such as SA‐β‐galactosidase, p16, p21 and Lamin B1 [19]. CD47 is a widely expressed transmembrane protein that act as a “don't eat me” signal to prevent inadvertent phagocytosis of normal cells by macrophages [20]. Evidence has been shown that aging is an important factor in controlling CD47 expression, and CD47 expression is elevated in diverse aged tissues and senescent cells [13]. More intriguingly, CD47 was recently identified as a hallmark of the dysfunctional aged muscle stem cells [16]. The present study showed that CD47 expression was significantly increased in the dermis of sun‐exposed aged skin samples, and this elevation was strongly associated with collagen reduction and elastin accumulation. In addition, we also demonstrated a positive relationship between the dermal expression of CD47 and p16, a classic senescence marker of photoaging [19]. These results suggest that dermal CD47 may be involved in the photoaging pathogenesis and serve as a potential senescence biomarker for evaluating the degree of photoaging.

Furthermore, we discovered that CD47 overexpressed in sun‐exposed aged dermis was predominantly localized in fibroblasts. As previously reported, CD47 could be upregulated in fibroblasts under a number of pathological states including scleroderma [21], pulmonary fibrosis [22] and aging [17]. In this study, we used normal human fibroblasts to construct a cellular photoaging model and confirmed the elevated expression of CD47 in the UVA‐induced photoaged fibroblasts. CD47 expression is known to be mediated by multiple mechanisms. The involvement of alternative splicing and post‐translational modifications such as ubiquitination in the regulation of CD47 expression is being revealed successively [23]. Moreover, transcriptional regulation of CD47 has been reported by factors including JUN, NF‐kB, and c‐Myc in the context of drug induction or gene mutation [21, 24]. As for UVA, the mechanisms responsible for its role in upregulating CD47 expression in fibroblasts during photoaging warrant further investigation.

We then proceed to probe the mechanism by which CD47 involved in photoaging. Recent evidence suggests that the senescent fibroblasts are accumulated in skin tissues during photoaging [5]. If the accumulated senescent fibroblasts are not cleared in time, they would spread senescence, impair ECM homeostasis, induce inflammation and stem cell exhaustion, thus forming a vicious cycle [25]. Immune system is responsible for eliminating senescent skin cells during aging to ensure skin tissue homoeostasis [11], and macrophages have been shown to clear senescent fibroblasts dependent on phagocytic function [26]. As a “do not eat me” signal, CD47 interacts with SIRPα on macrophages to inhibit phagocytosis, which is often hijacked by senescent cells to evade macrophage surveillance [13]. For instance, the apoptotic senescent osteoblasts were found to highly express CD47, allowing them to escape from osteoimmune clearance by macrophages and accumulate in bone marrow [15]. These reports led us to wonder whether the photoaged fibroblasts with high CD47 status evade elimination by macrophages in a CD47/SIRPα dependent manner during photoaging. In this work, we demonstrated that photoaged fibroblasts could be phagocytosed by macrophages, but impaired the phagocytotic function of the co‐cultured macrophages via CD47/SIRPα axis. Moreover, blocking CD47/SIRPα axis was found to improve the ability of macrophages eliminating the photoaged fibroblasts. These findings indicated that the CD47/SIRPα axis activation inhibits macrophage‐mediated phagocytosis to hinder the elimination of photoaged fibroblasts. In line with previous studies [5], we also observed increased numbers of senescent fibroblasts accumulated in the sun‐exposed skin tissues. More importantly, the majority of accumulated senescent fibroblasts was CD47 positive, which was accompanied by the elevated numbers of SIRPα^+^ macrophages. These histological observations further provided supportive evidence for the participation of the CD47/SIRPα axis in senescent fibroblasts accumulation during photoaging. In the next phase, we will conduct in vivo experiments to further validate the role of CD47/SIRPα axis in photoaging. Actually, beyond the CD47/SIRPα phagocytosis checkpoint, aging‐related defects in macrophage phagocytosis could be triggered by SASP [27], immunosenescence [27], oxidative stress [28] and so on. Hence, there might be other mechanisms that contributes to the impaired elimination of photoaged fibroblasts by macrophages, which should be further explored in the future. What's more, besides its canonical “don't eat me” signal function, CD47 has been shown to exert direct regulatory effects on numerous cellular behaviors and functions [29]. To further our understanding of CD47's pathogenic roles in photoaging, we will study its direct influence on fibroblasts in further investigations.

In conclusion, the present research revealed the potential of dermal CD47 as a novel biomarker for assessing photoaging. Furthermore, we identified the role of CD47/SIRPα axis in senescent fibroblasts accumulation during photoaging through inhibiting macrophages‐mediated elimination, which might be a potential target to intervene in photoaging.

## Author Contributions

Xinya Xu performed experiments and wrote the manuscript; Xinhua Lu collected and analyzed data and wrote the manuscript; Xinling Chen and Amin Yao performed experiments; Xinling Chen collected clinical samples and clinical data; Wei Lai designed and supervised the study, and revised the manuscript. All of the authors have read and approved the submitted version of the manuscript.

## Ethics Statement

The study was conducted according to the guidelines of the Declaration of Helsinki and was approved by the institutional review board (approval no: [2023] 02‐266‐01).

## Conflicts of Interest

The authors declare no conflicts of interest.

## Data Availability

Data sharing not applicable to this article as no datasets were generated or analysed during the current study.
